# Evaluation of a knowledge-attitude-practice model based narrative life education program for community-dwelling older adults: a mixed-methods feasibility study

**DOI:** 10.1186/s12877-024-05153-4

**Published:** 2024-06-24

**Authors:** Xifeng Xie, Li Zhou, Xiaoling Zhang, Huina Zou, Yuanfeng Lu, Huimin Xiao

**Affiliations:** 1 School of Nursing, Fujian Medical University, Fuzhou City, Fujian Province China; 2 Nanjie Community Health Service Center, Fuzhou City, Fujian Province China; 3 Research Center for Nursing Humanity, Fujian Medical University, Fuzhou City, Fujian Province China

**Keywords:** Older adults, Knowledge-Attitude/Belief-Practice theory, Narrative, Death education, Feasibility study

## Abstract

**Background:**

The global aging population presents challenges that are particularly acute in China. Older Chinese adults’ attitudes towards death significantly impact their quality of life. Death education is crucial for promoting positive perspectives on life and death. Narrative education offers a promising approach to facilitating death education. Integrating the Knowledge-Attitude-Practice (KAP) model into death education will enhance the feasibility and acceptability of death education programs.

**Methods:**

This mixed-methods feasibility study included a quasi-experimental trial and semi-structured interviews. Older adults in the intervention group (*N* = 27) received a 6-week KAP-based narrative life education program in addition to standard community health education; participants in the control group (*N* = 20) received only the normal community health education. In both groups, attitudes toward death and the meaning of life were assessed at baseline and immediately after the intervention. A post-intervention semi-structured interview and satisfaction survey were also conducted for the intervention group.

**Results:**

Forty out of 47 older adults completed the program for an 85.1% retention rate. All of the older adults in the experiment were very satisfied and satisfied with the life education program, and no adverse events were reported. Compared to the control group, participants in the intervention group had a significant decrease in the fear of death (*P* = *0*.028), and substantial improvement in their value of life (*P* = *0*.031), goal of life (*P* = *0*.035), freedom of life (*P* = *0*.003), and the total score for purpose in life (*P* = *0*.017). The qualitative results yielded four themes: profound recognition of life and death, contradiction between thoughts and action, conflict between one’s acceptance and others’ avoidance, and evaluation of the life education program.

**Conclusions:**

The KAP-based narrative life education program is feasible and acceptable for older Chinese community-dwelling adults. It is also potentially effective in improving attitudes toward death attitudes and the meaning of life in this cohort.

**Trial registration:**

This study was retrospectively registered at China Clinical Trial Registry as ChiCTR2300069551 on 2023-03-20. URL of registration: https://www.chictr.org.cn/showproj.html?proj=183176.

**Supplementary Information:**

The online version contains supplementary material available at 10.1186/s12877-024-05153-4.

## Background

The elderly population is poised to significantly increase around the world. By 2050, adults over 65 are projected to account for 16% of the global population, with the proportion of individuals aged 60 and above in China likely reaching 35% [1, 2]. Death is inevitable, but it is a sensitive topic, especially in China. Older adults are prone to being increasingly aware of death due to their decline in physical function, the threat of chronic disease, and an increased witnessing of their peers’ deaths [3].

Attitudes towards death are not only related to the physical and mental health of older adults, but also affect their preparation for death and quality of life beforehand [4]. Ignorance of death preparation increases fear and anxiety about death [5]. However, a deep-rooted traditional culture that, emphasizes life and neglects death has made death a taboo subject in China. It is not easy for most Chinese people to communicate about death-related issues [6]. Although older adults can accept death as a part of life, most of them still feel fear and avoid talking about death-related topics [7]. Compared to nursing home residents, community-dwelling older adults are more afraid of facing death and feel it is more difficult to deal with life-and death-related issues [8]. Thus, further exploration is needed regarding how to help Chinese older adults establish a reasonable understanding of and attitudes toward life and death.

The essence of life education for older adults is orientation regarding the subjects of life and death, with death education comprising the core content [9]. The program teaches individuals how to recognize and face death [10]. The goal is to facilitate acceptance of end of life, process of death, and experience of bereavement in terms of the individual’s knowledge, attitudes, and skills [11, 12]. Previous studies have shown that death education promotes positive changes in death-related attitudes, enhances the sense of meaning in life, and improves the quality of life [13–15]. However, previous programs have mainly focused on the stages of life and meaning of death and failed to address cultural conflicts in the process of death education, which may result in participants’ psychological discomfort. Thus, developing death education programs have been proposed that operate from the perspective of life’s course in order to reduce negative emotions and the fear of death [16]. Given the sensitivity of the topic, the method of delivering such education must be carefully considered.

Narrative education is an approach to achieving educational and research purposes by narrating, explaining, and reconstructing the experiences of educators and participants [17]. When addressing a sensitive topic, narratives generate less resistance because of the storytelling model [18]. Narratives may also facilitate older adults establishing reasonable cognition, knowledge, and behaviors related to death through introspection regarding their experiences and creation of meaning in their lives [19]. Therefore, the method can be used in death education in older adults [20, 21].

A theoretical model is critical for framing the program, guiding data collection, and interpreting findings [22]. Various death education models have been developed such as lecture teaching and experience-sharing models [16, 23]. In fact, compared to non-narrative messages, messages in narrative education have a stronger persuasive impact on one’s attitudes, intentions, and behaviors, both immediately and over time [24]. Therefore, a model that comprehensively attaches information acceptance, attitude modification, and behavior transition should be employed. The theory of Knowledge-Attitude-Practice (KAP) was first proposed by Cust and Mayo to explain the progressive relationship of moving from knowledge acquisition to behavior modification in individuals [25]. With the goal of helping individuals establish positive attitudes and beliefs and shifting towards correct behavior based on the reception and mastery of relevant knowledge, the theory has been widely applied in predicting health-related behaviors and implementing practice-improvement programs [26]. However, few studies have integrated the KAP model into death education for older adults, though it has the potential to communicate essential information, achieve reasonable life and death cognition, facilitate the maintenance of a positive attitude, and encourage the development of death-coping strategies [27]. Therefore, this study aimed to develop a KAP-based narrative life education program and explore its feasibility and effects on attitudes toward death and sense of meaning of life in older community-dwelling adults.

## Methods

### Study design

This mixed-methods feasibility study involved a quasi-experimental trial and semi-structured interviews. The goal was to determine the feasibility, acceptability, and primary efficacy of a narrative death education program for community-dwelling older adults. This study was reported following the Mixed Methods Reporting in Rehabilitation & Health Sciences (MMR-RHS) and was performed in accordance with the Declarations of Helsinki [28].

### Setting and sample

From September to November 2022, older adults were recruited from a community located in Fuzhou City, China, from September to November 2022. It home to approximately 4,500 individuals aged 60 and above, constituting more than 19.90% of the total residential population. The inclusion criteria were: (a) aged 60 years and above and (b) able to understand and communicate in Chinese. The exclusion criteria were: (a) with cognitive impairment or (b) with severe visual, auditory, or mental disorders. A sample size between 24 and 50 participants is recommended for feasibility studies [29]. A total of 47 community-dwelling older adults were recruited for this study. Details of recruitment are in Additional file 1.

### Recruitment

Participants were recruited at the community health service center via two approaches. For on-site recruitment, a recruitment poster was posted at the center. Potential participants who were interested in the study could directly contact the research assistant (RA). The RA then introduced this study to them through a face-to-face interview. For tele-recruitment, the RA interviewed potential participants via telephone, based on a list of older adults provided by the center. Written informed consent was obtained from each participant. After baseline data collection, individuals were invited to voluntarily join either the intervention or control group according to their preferences. Given the sensitivity of the topic of death among Chinese older adults, we did not employ randomization.

### Intervention program

Both groups received the usual health education provided by the community health center. The intervention group also received the KAP-based Narrative Life Education (KAPNLE) program.

### Intervention group

The KAPNLE program was initially drafted after reviewing KAP theory and multimedia material on life education developed by the research team and engaging in internal brainstorming. Details of the program were revised according to two rounds of comments from a six-expert panel whose research areas involved geriatric nursing, life education, community nursing, psychology, and social work. We also conducted interviews with five community-dwelling older adults and further refined the program based on their feedback.

The final version was composed of four modules: Understanding Life and Death (Knowledge), Viewing Life and Death (Attitude), Preparing for Death (Practice), and Transcending Life and Death (Practice). These four modules covered a total of six sessions, including *Life Course*, *Growing Old Peacefully*, *Passing Away in Pain*, *Saying “Goodbye” Well*, *Expressing “Love”*, and *Living a Wonderful Life*. Each session was conducted according to a four-step narrative process by a researcher who served as the facilitator. Initially, the facilitator presented material on life and death issues and created a context within which participants could easily discuss topics of life and death. Participants were then invited to redescribe the topics in their own way and share their impressions. Next, they were guided to further reflect on their own experiences and discuss views on issues related to life and death. In the final step, activities on related themes were conducted in a relaxed environment to deepen participants’ knowledge and experience of life and death. The program was conducted once a week over six weeks and lasted 30 to 60 min per session. It was held offline in the visiting room of the community healthcare center and attended by groups of six or seven older adults. The details of the program are shown in Additional file 2.

### Control group

The control group met twice a month and only received the usual community health education, which includes topics related to chronic diseases, medication safety, and lifestyle management, their course did not involve life education.

### Measurements

#### Basic information questionnaire

Demographics and information regarding life and death issues were collected by a self-reported questionnaire designed by our research team (Additional file 3). The demographic information included age, gender, religion, education level, marital status, living status, and number of children. The issues related to life and death included life-threatening illness experiences, self-perceived physical health, most profound encounters with death, and communication about death topics.

### Death Attitude Profile-Revised (DAP-R)

Death attitudes were assessed using the Chinese version of Death Attitude Profile Revised (DAP-R) [30]. It consists of 32 items and five dimensions: fear of death, death avoidance, neutral acceptance, approach acceptance, and escape acceptance. Each item is rated on a five-point Likert scale ranging from 1 (strongly disagree) to 5 (strongly agree). Death attitudes are judged by the scores for each dimension. The higher the score, the more inclined the respondent is to this dimension’s attitude. In Chinese older adults, the Cronbach’s α values for the five dimensions were 0.796, 0.670, 0.621, 0.842 and 0.771.

### Purpose in Life Test (PIL)

Meaning of life was measured using the Chinese version of Purpose in Life Test (PIL) [31]. It contains 20 items across four dimensions: quality of life, value of life, goal of life, and freedom of life. Scores are assigned using a five-point Likert scale, with each dimension including positive and negative questions. Scores selected for negative questions are reversed. Higher total scores indicate a greater sense of meaning of life. The Chinese version of the PIL has been validated, with a Cronbach’s α of 0.878.

### Satisfaction of the program questionnaire

Respondents’ satisfaction was assessed using a self-designed questionnaire with six items: education theme, education content, education form, education schedule, benefits and practicability, and overall satisfaction. Each item is rated from “strongly satisfied” to “strongly dissatisfied” (Additional file 4).

### Semi-structured interview

To assess the feasibility of the program for Chinese older adults, we conducted semi-structured interviews with the intervention participants. The interview outline was developed by the research team and began with a primary open-ended question: “What are your perceptions of the KAPNLE?” This question allowed participants to freely express their feelings and feedback about the program. Probing questions were then asked to facilitate in-depth exploration. The interview guideline is shown in Additional file 5.

### Data collection

The quantitative data were collected by another trained RA, who was blind to the group assignments. All participants’ death attitudes and ideas regarding the meaning of life were assessed at baseline and immediately after the program. In addition, the experimental participants were invited to describe their satisfaction with the program.

Participants in the intervention group were interviewed about their perceptions and experiences immediately after the program. A one-on-one semi-structured interview of each was conducted by the RA in the visiting room of the community healthcare center. Each interview lasted about 30 to 45 min, and the content was recorded with the participants’ informed consent.

### Data analysis

#### Quantitative data

The quantitative data were analyzed using IBM SPSS 27.0. The data were normality tested before being analyzed. Mean and standard deviation, median (P_25_, P_75_), number, and percentage were determined to describe the older adults’ characteristics. A Chi-square test, t-test, Fisher’s Exact Test, Wilcoxon rank sum test, and multiple regression were used in this study. The Wilcoxon rank sum test was employed to test differences in the attitudes toward death and meaning of life between the groups for data with abnormal distributions. Multiple regression analysis was used to adjust for baseline imbalances in attitudes toward death and meaning of life between the two groups. Line graphs were used to describe any changes.

#### Qualitative data

The interviews were transcribed verbatim by a researcher within 24 h of their being conducted. Qualitative content analysis was used to analyze the qualitative data [32]. The steps applied were as follows: (a) identify and segment meaningful sentences within each interview text to generate “meaning units”, (b) condense semantic units into “condensed meaning units”, (c) abstract condensed semantic units to generate “codes”, (d) compare codes for commonalities, categorize codes into “categories”, (e) discuss and consensus on categories and formulate “themes” (Table 1). The interview data were coded by two researchers working independently. To ensure the credibility of the results, we used peer debriefing, member checks and held regular meetings to discuss the data analysis process and inconsistent opinions.

**Table 1:** Example of the qualitative data analysis

(a)Meaning units	(b)Condensed meaning units	(c)Codes	(d)Categories	(e)Theme
When you asked me about life and death, I really didn’t know how to answer. I never thought about it before. Life means I’m still alive; and death means I am away from the world.	- uncertainty regarding the question of life and death	- uncertainty of life and death	Vague concept of life and death at the early stage	Profound recognition of life
	- lack of prior consideration of life and death issues	- no prior consideration		
	- association of life with being alive and death with departure from the world	- simple recognition of life and death		
I feel that my life is a line with ups and downs, starting from zero, maybe ending at 100. Each number represents a stage of my life, and contains many important things.	- Recognize life is a progressive journey and each stages have important events	- Emerging personal understanding of life	Gradually clarifying life and death issues	

## Results

### Feasibility of the program

A total of 47 older adults were recruited, including 27 in the intervention group and 20 in the control group. Seven in the intervention group withdrew from the study. Therefore, a total of 40 individuals completed the follow-up measurement and were included in analysis (Additional file 3). The total retention rate was 85.1%. Sixteen participants in the intervention group finished four to six sessions of the program, while four participants missed three out of six sessions. Their reasons included physical illness, family members’ illness or death, schedule conflicts, and self-isolation due to COVID-19 infection. The completion rate was 80.0% (16/20). All 20 intervention participants were very satisfied or satisfied with the education program, including its modules, sessions, implementation theme, and overall participation experience. No adverse events were reported during the study.

### Participant characteristics

The mean age of the participants was 73.33 (6.16) years, with ages ranging from 63 to 88 years. Most of them were women (62.5%), not religious (75.0%), married (75.0%), had a high school education or above (75.0%), had one child (62.5%), lived with their children or spouse (85.0%), perceived themselves as in general or poor physical health (65.0%),were moved by their parents’ death (72.5%), and never communicated about death (62.5%). A small percentage had suffered (22.5%) or had family member who suffered (15.0%) from a life-threatening disease. There were no significant differences in demographic characteristics between the two groups (Additional file 6).

### Preliminary efficacy of the program

#### Death attitudes

Compared to the control group, a significant decrease was observed in fear of death in the intervention group (*P* = 0.028), no significant differences were detected in the other dimensions or total score of the DAP-R (*P* > 0.05). However, upward trends were observed in the DAP-R’s natural acceptance and approach acceptance. For the dimensions of death avoidance and escape acceptance, slight changes could also be found. After the intervention, there was no significant difference in total score of the DAP-R between the two groups, but the score for the control group fluctuated greatly during the follow-up.

#### Meaning of life

Compared to the baseline measurement, there were greater increases in value of life, goal of life, freedom of life, and total score of the PIL for the intervention group (*P <* 0.05), and a slight upward trend was observed for freedom of life. However, no significant difference in the PIL’s quality of life was found between the two groups (*P* = 0.141). As shown in the line chart, differences were observed in the trends in the total scores for the PIL between the two groups, after the intervention, the scores for the intervention group increased markedly compared to the control group after the intervention (Additional file 7 and Additional file 8).

### Perceptions of the program

According to the post-intervention interview, four themes and ten sub-themes were identified: (a) profound recognition of life, (b) contradiction between thoughts and action, (c) conflict between self-acceptance and others’ avoidance, and (d) evaluation of the life education program.

#### Profound recognition of life

This theme relates to older adults’ cognition of life and death, and contains three sub-themes:

#### Vague concept of life and death at the early stage

Some respondents stated that they had a vague understanding of life and death at the beginning of the program and had difficulties in describing or explaining them. They also expressed that they paid no attention to life and death-related issues in their daily lives over in years past.

*“When you asked me about life and death, I really didn’t know how to answer. I never thought about it before. Life means I’m still alive; and death means I am away from the world. Is it right?” (Participant 2)*.

#### Gradually clarifying life and death issues

After the first two sessions of the program. participants expressed that they had a figurative understanding of life and death. They realized the logical relationship between the two, and further accepted their unique lives.

*“I didn’t know what life was like before, but now I do. My life is like a sunflower gone to seed. If I pass away like a flower withers, I still have something left in this world.” (Participant 1)*.

*“I feel my that life is a line with ups and downs, starting from zero, maybe ending at 100. Each number represents a stage of my life, and contains many important things.” (Participant 10)*.

#### Discussing life and death with an open mind at the final stage

At the end of the program, participants expressed that they could easily discuss topics related to life and death and felt comfortable in the process. They also said that the program reduced their negative feelings about death and encouraged them to pursue meaning in their lives.

*“Now I have a new perspective on life, and the fears about death seem to have vanished. Dying at the age of 20 or 100 are both lifetimes and being dead or alive cannot be decided by oneself. So, I will cherish my life when I am alive and enjoy life every day.” (Participant 11)*.

*“I realized that talking about death is not as difficult as I imagined. It actually could be very easy, just like this program.” (Participant 6)*.

#### Contradictions between thoughts and actions

This theme is related to inconsistencies between positive thoughts about life and death and passive behaviors regarding death preparation, it includes the following two sub-themes:

#### Hold positive thoughts on to embrace life and deaths

Some participants expressed that they held rational and open attitudes about life and death after the program. They realized the inevitability of death, and calmly accept it as a normal phenomenon.

*“I think that everyone will die in the end, and nobody can avoid death. I must go on my last journey well. As long as I have done all things, I can go without any regret.” (Participant 12)*.

*“I never thought about it (death) until I participate in this program. It reminded me that I would pass away one day. Then, I started to think about death issues in advance. If I had not participated in it, I wouldn’t have come to this step.” (Participant 5)*.

#### Hesitating to make life and death plan

Some of the older adults emphasized living in the present, and were unwilling to make death preparation in advance.

*“I am not thinking about what I should do about death at present. I just want live in the moment, do what I need to do at present, and stay happy.” (Participant 7)*.

*“I will think about these things, such as the cemetery or family arrangements, when I am more than 70 or 80 years old. But now, I live in the present and enjoy life.” (Participant 8)*.

#### Conflict between one’s acceptance and others’ avoidance

This theme is related to the acceptability of life education, two sub-themes comprise this category.

#### Self-acceptance and openly discussion about life and death

Some participants noted that the program changed their attitudes about life and death. They not only felt comfortable talking about death-related topics, but also recommended the program to others.

*“I think this program should be recommended to more older adults, especially to those who are sensitive and concerned about death. It can teach them how to deal with life and death, and overcome fear of death.” (Participant 10)*.

#### Others’ opposition to life education due to stereotypes

Some participants mentioned that their family members or friends opposed their participation in the life education program due to the sensitivity of death-related topics.

*“Most older adults around me resist talking about death. They tried to persuade me not to participate in life education because death is a taboo.” (Participant 4)*.

*“My family didn’t want me to attend such activity, for it will bring bad fortune. Therefore, I attended this class without telling my family.” (Participant 13)*.

#### Evaluation of the life education program

This theme is related to the participants’ perspectives on the program and includes two sub-themes:

#### Affirmation of the program

Some of the older adults reflected that conversations about life and death were sensitive but acceptable. They further expressed that they could benefit from life education.

*“It is not easy to talk about death-related topics, but I think life education is very important. Because it can help older adults do enough preparation and pass away without any regrets. I support this program.” (Participant 3)*.

Some participants believed that the program was feasible because narrative life education enabled them to pick up death topic more easily. Moreover, the program was conducted in groups, which established a supportive environment.

*“The most impressive thing about this program is telling stories. I have received various stories from others. Then, I felt pleased to share my thoughts and discuss with others about these stories.” (Participant 15)*.

*“It was easier for me to talk about life and death in groups. When someone started to talk about that, then we thought we could talk about that as well. You know, such an environment is important.” (Participant 17)*.

#### Comments and suggestions

Some of the respondents mentioned that the group-based education might ignore individuals’ specific needs and suggested combining group education with individual counseling in the future.

*“There was often someone absent in the group. Therefore, I think the program could add some individual education content, which would help the absentee to catch up with the progress.” (Participant 10)*.

Regarding the resistance of their family or close friends to this program, some of the older adults hoped that the program could expand to include outside participants, such as by allowing them to invite people around them to participate.

*“In fact, I still hope to get support and understanding from my family or friends, so maybe you can try to invite them to participate in this life education together.” (Participant 4)*.

## Discussion

To the best of our knowledge, this is the first study to develop and evaluate the feasibility and preliminary effects of the KAPNLE. Our quantitative findings demonstrate that the program is effective at promoting a positive transition in death attitude and improving the meaning of life for community-dwelling older adults. The qualitative results indicate that the program is both acceptable and feasible. It also supports the potential of using the KAPNLE to change people’s attitudes toward life and death.

Our study indicates an acceptable feasibility among older adults. Seven participants withdrew from the study (a dropout rate of 14.8%), which was higher than a previous study [33]. One possible reason for the dropout rate may be that the sensitivity of the topic may have negatively affected these Chinese older adults’ willingness to participate [34]. Additionally, the study was conducted during the period of the COVID-19 pandemic, when older adults were more concerned about their physical health and less likely to engage in social activities [35]. There were no reported adverse events during the study for the intervention group, and all participants were either very satisfied or satisfied with the KAPNLE, indicating that the program is acceptable and safe. The KAP theory focuses on shared goals, transparency, accountability, and respect, and these factors are essential for effective collaboration in patient engagement [36]. Based on group-formatted discussions, the contents of the KAPNLE programs matched knowledge to attitudes, and then to action, in sequence rather than in a fragmented fashion. Participants’ feeling of freedom was encouraged at every step to promote their acceptance of death education. The older adults participating in our study stated that narration helped them address topics of life and death more easily and discuss related issues with less emotional resistance. In fact, the narrative approach emphasized finding psychosocial strengths and highlighting their own personal meaning of life [37]. Compared to didactic messages, those in narrative form tend to be more acceptable due to their natural format and the emotional engagement, and positive thoughts they inspire [38]. The narrative method can provide rich insights into the meanings associated with phenomena due to its deep subjectivity and inherent explanations of information [39]. Based on the multimedia cases we provided, the KAPNLE program adopted a four-step group-based narrative process that created a relaxed and supportive environment by focusing on storytelling, thus promoting acceptance of issues related to life and death [40]. This approach is useful in helping older adults understand and reconsider events in their lives, encouraging them to share information and transform their attitudes about death in an acceptable way [41].

Our quantitative results demonstrate that it is feasible to use the KAPNLE to promote a positive transition in attitudes toward death, especially in terms of reducing the fear of death, and these results are consistent with previous studies [33, 42]. The post-program qualitative interviews indicated similar findings. The program provided relevant information about the process of life, hospice care, living wills, and death preparations. Correlating with KAP theory, the provision of comprehensive information about death can promote a more positive attitude, which can then help bridge the knowledge-intention gap in discussions about and preparations for death [43]. Moreover, participants also disclosed that the program had a promising effect on their understanding of the meaning of life and improved their acceptance of death. The KAPNLE guided these older adults to realize the inevitability of death through free discussion in a non-didactic group format and relaxing circumstance. Once their cognition of death was modified, they may experience greater openness to the possibilities available to them throughout the rest of their lives [44]. In line with previous studies, our results suggest that the KAPNLE improves the sense of meaning of life in older adults [45, 46]. Participants were guided to be more aware of their achievements and self-value by reviewing their lives, and resist the consciousness of death by perceiving and maintaining a positive sense of self-meaning [47]. In line with concepts of KAP theory, modification of the cognition of and attitudes about death plays a crucial role in clarifying the meaning of life, finding purpose in life, and identifying suitable coping strategies [48].

Although the program encouraged participants to set goals and make plans for the rest of their lives, no direct changes in participants’ behavior were observed. The behaviors related to death preparation among older adults are often affected by family circumstances and subjective norms, and it is challenging to establish advanced death preparation due to the Chinese culture [49, 50]. In addition, health status is associated with death preparation in older adults. Most participants in our study were not facing life-threatening illnesses, which may have reduced their initiative to engage with this program [51].

This study has some limitations, because of the cultural influences and stereotypes of death education from older adults’ family members, the dropout rate was relatively high. Moreover, although most participants had accepted the life process and found the meaning of life, they remained negative about making pre-death plans. This indicates that some of them may not have been operationally prepared for death and thus could not achieve the behavioral transition envisioned by the KAP model. Some participants also suggested that their family members or acquaintances should be involved in this program. In fact, due to the sensitivity of discussing about death within the traditional views of Chinese older adults, we allowed participants to choose group assignment by themselves to prevent ethical conflicts. This probably introduced selection bias for participants who chose to receive the intervention may have already been more open-minded about discussing death. In addition, this study did not employ a randomized controlled trial because our purpose was to explore the feasibility and acceptability of the program. Further research with rigorous design is needed to enhance participants’ adherence to narrative death education and to optimize intervention strategies.

## Conclusion

This study constructed a KAP-based narrative life education program for community-dwelling older adults. We found that the life education program is acceptable and feasible among this cohort and could potentially improve attitudes toward death and the meaning of life. Future research with a rigorous design is necessary to test the effectiveness of narrative death education in older adults.

### Electronic supplementary material

Below is the link to the electronic supplementary material.

Supplementary Material 1

## Data Availability

The datasets used and/or analyzed during the current study are available from the corresponding author upon reasonable request. The data are not publicly available due to privacy and ethical restrictions.

## Abbreviations

KAP: Knowledge-Attitude-Practice
KAPNLE: KAP-based Narrative Life Education
RA: Research assistant
DAP-R: Death Attitude Profile-Revised
PIL: Purpose in Life Test
