# Supplementary material for: Evaluation of a knowledge-attitude-practice model based narrative life education program for community-dwelling older adults: a mixed-methods feasibility study
Source: BMC Geriatr. 2024 Jun 24;24:547. doi: 10.1186/s12877-024-05153-4 (PMC11194897; doi:10.1186/s12877-024-05153-4)
Supplement: Supplementary file 1 — Supplementary Material 1 [file 12877_2024_5153_MOESM1_ESM.docx]

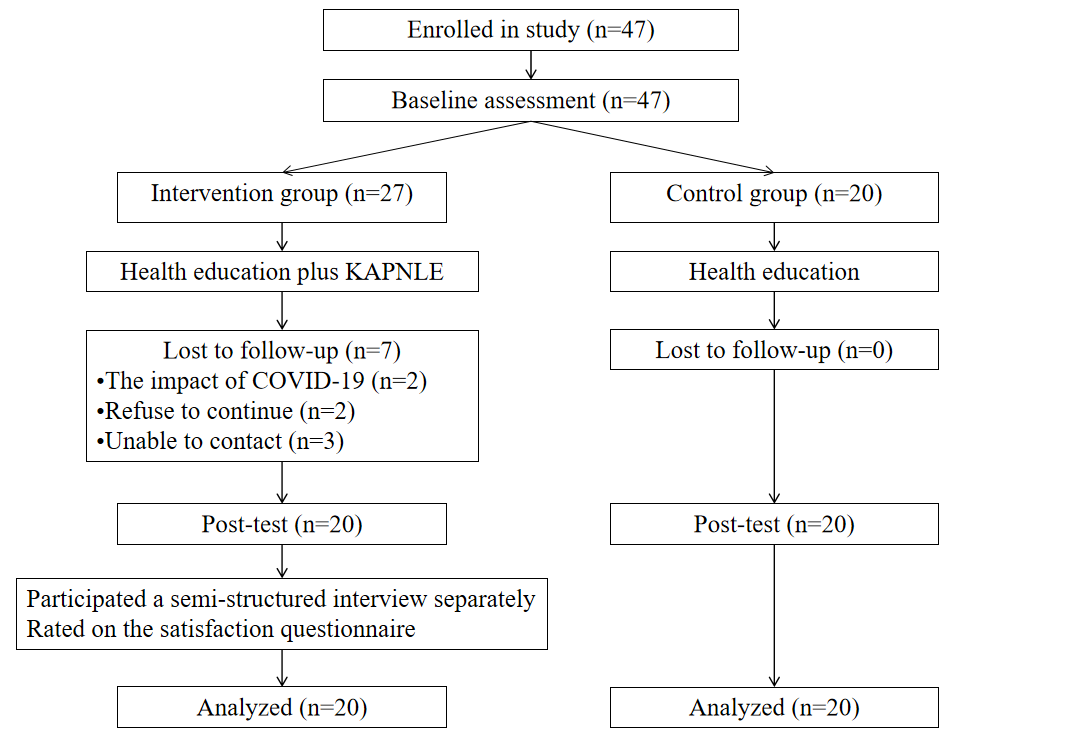


Additional file 1 Study flow diagram

Additional File 2 Main Contents of The Life Education Program.

| Module | Session | Theme | Exemplary task or exercise | Tools |
| --- | --- | --- | --- | --- |
| Understanding Life and Death | 1 | Life Course | •Review six stages of life with twelve important life events  •What is the end of life and what do you think about it?  •Draw an up-and-down line based on important life events | Video "0-100 Years Old Chinese"  Guiding questions  My Lifeline |
| Viewing Life and Death | 2 | Growing Old Peacefully | •Tell us your impressions of the video  •Share your life-death views with others  •How much do you know about hospice care?  •Choose and color a representational portrait of life | Video "Tomb-Sweeping Day, Let's Talk about Views on Life and Death"  Guiding questions  Color for life |
|  | 3 | Passing Away in Pain | •What do you think death with dignity is?  •How much do you know about advance medical directives?  •Make a safeness pendant by hand | Story "Life's Choices"  Guiding questions  Add happiness to life |
| Preparing for Death | 4 | Saying Goodbye Well | •What is the death preparations’ contents?  •List what you have prepared for death  •Write notes to express "thank" and "apologize" to family | Picture book "Goodbye, Grandma Emma"  Guiding questions  Happy Message |
|  | 5 | Saying Love | •Tell us your impressions of the video  •Tell us your enlightenment from the video  •What is the meaning of death preparation?  •Write notes to express "love" and "goodbye" to family | Video "Don't Let Love be so Tangled at the end of Life"  Guiding questions  Happy Message |
| Transcend Life and Death | 6 | Living More Wonderful Life | •List the life-threatening things you ever experienced  •What is the meaning and the value of your life?  •Write down your unfinished wishes and future planning | Video "You Make Life Spring"  Guiding questions  Wish List |

Additional file 3 Basic information questionnaire

**1. Age (years):**  ______

**2. Gender:**  Male Female

**3. Ethnicity:** Han Other _______

**4. Educational level:**
 Elementary school or below Junior high school

Senior high school or technical secondary school College or undergraduate

Postgraduate or above

**5. Marital status:**
 Single Married Divorced Widowed

others (please specify: ______ )

**6. Living status:**

Living with spouse Living with children

Living with spouse and children Living with relatives

Living alone Other

**7. Number of children:**
 0 1 2 3

More than 3 (please specify: _______ )

**8. Do you hold any religious beliefs?**
 Yes (please specify: _____ ) No

**9. Have you ever suffered from life-threatening diseases over the past years?**

Yes (please specify:______ ) No

**10. How do you perceive your current health status?**
 Very good Good Medium Poor Very poor

**11. Is there any family member in your household currently suffering from life-threatening diseases?**
 Yes (please specify: ______ ) No

**12.** **Whose passing has left the deepest impression on you?**
 Parents Spouse Siblings

Other (please specify:______ )

**13. Which of the following factors most influences your attitudes towards death?**
 Religious beliefs Books and magazines

Elders’ attitudes towards death in the family Funerals attendance

Personal reflection and exploration Personal health status

Death of relatives or friends Radio or movies

Other (please specify: ______ )

**14. Have you ever discussed death related topics with others?**
 Yes No

**15. What is the atmosphere like when you discuss death with others?**
 Open and natural Avoid discussing as much as possible
 Relaxed but ends quickly Relaxed and discussed thoroughly
 Awkward and ends quickly Awkward but fully discussed
 Only mentioned when necessary, and avoiding those who do not want to know

Other (please specify:______ )

Additional file 4 Satisfaction of the Program Questionnaire

To understand your satisfaction with this program, please mark next to the option that you feel best represents your opinion.

| **Characteristics of the program** | **Very Satisfied** | **Satisfied** | **Neutral** | **Dissatisfied** | **Very Dissatisfied** |
| --- | --- | --- | --- | --- | --- |
| Theme |  |  |  |  |  |
| Content |  |  |  |  |  |
| Form |  |  |  |  |  |
| Scheduling |  |  |  |  |  |
| Benefit and practicality |  |  |  |  |  |
| Overall satisfaction |  |  |  |  |  |

Additional file 5 The interview guide

- What are your feelings during the process of participating in this program?
- What positive or negative impacts did the program have on you? or have you experienced any changes after participating in this program? What are they?
- What do you think about the program in terms of the time schedule, format, and content of the program?
- What attracted you to participate in this life education program?
- Were there any parts that affected your enthusiasm?
- What was the most memorable content for you? What insights did it bring you?
- How necessary do you think life education are for the elderly? Why?
- What do you think are the shortcomings of this program that need improvement, or do you have any suggestions?
- Will you be willing to participate in similar activities in the future? Why?

Additional file 6 Participant characteristics and group comparisons.

| Characteristics | Total  (N=40) | | Intervention group(N=20) | | Control group  (N=20) | | *t*/*χ*^2^  (*P*) | |
| --- | --- | --- | --- | --- | --- | --- | --- | --- |
| **Age (years)** |  | |  | |  | | 0.668^c^ | |
| Mean (SD) | 73.33(6.16) | | 73.75(5.62) | | 72.90 (6.78) | |  | |
| Range | 63-88 | | 65-83 | | 63-88 | |  | |
| **Gender, n (%)** |  | |  | |  | | 0.327^a^ | |
| Men | 15(37.5) | | 6(30.0) | | 9(45.0) | |  | |
| Women | 25(62.5) | | 14(70.0) | | 11(55.0) | |  | |
| **Religion, n (%)** |  | |  | |  | | 1.000^a^ | |
| Yes | 10(25.0) | | 5(25.0) | | 5(25.0) | |  | |
| No | 30(75.0) | | 15(75.0) | | 15(75.0) | |  | |
| **Education, n (%)** |  | |  | |  | | .440^a^ | |
| ≤Primary school | 3(7.5) | | 2(10.0) | | 1(5.0) | |  | |
| Junior high school | 7(17.5) | | 3(15.0) | | 4(20.0) | |  | |
| High school | 18(45.0) | | 11(55.0) | | 7(35.0) | |  | |
| College | 12(30.0) | | 4(20.0) | | 8(40.0) | |  | |
| **Marital status, n (%)** | |  | |  | |  | 0.144^a^ |  |
| Married | | 30(75.0) | | 17(85.0) | | 13(65.0) |  |  |
| Single/divorced/widowed | | 10(25.0) | | 3(15.0) | | 7(35.0) |  |  |
| **Living status, n (%)** | |  | |  | |  | 0.182^b^ |  |
| Spouse or Children | | 34(85.0) | | 19(95.0) | | 15(75.0) |  |  |
| Alone | | 6(15.0) | | 1(5.0) | | 5(25.0) |  |  |
| **Number of children, n (%)** | |  | |  | |  | 0.927^a^ |  |
| 1 | | 25(62.5) | | 12(60.0) | | 13(65.0) |  |  |
| 2 | | 9(22.5) | | 5(25.0) | | 4(20.0) |  |  |
| ≥3 | | 6(15.0) | | 3(15.0) | | 3(15.0) |  |  |

*Note.* a, *χ*^2^ test; b, Fisher's exact test; c, t-test; SD, Standard deviation.

Additional file 6 Participant Characteristics and Group Comparisons (Continued).

| Characteristics | Total  (N=40) | Intervention group(N=20) | Control group  (N=20) | *t*/*χ*^2^  (*P*) |
| --- | --- | --- | --- | --- |
| **Ever suffered from a life-threatening disease, n (%)** |  |  |  | 1.000^b^ |
| Yes | 9(22.5) | 4(20.0) | 5(25.0) |  |
| No | 31(77.5) | 16(80.0) | 15(75.0) |  |
| **Family member ever suffered from a life-threatening disease, n (%)** |  |  |  | 1.000^b^ |
| Yes | 6(15.0) | 3(15.0) | 3(15.0) |  |
| No | 34(85.0) | 17(85.0) | 17(85.0) |  |
| **Self-perceived physical health, n (%)** |  |  |  | 0.470^a^ |
| Very well | 2(5.0) | 2(10.0) | 0(0.0) |  |
| Well | 12(30.0) | 5(25.0) | 7(35.0) |  |
| Common | 19(47.5) | 9(45.0) | 10(50.0) |  |
| Poor | 7(17.5) | 4(20.0) | 3(15.0) |  |
| Very poor | 0(0.0) | 0(0.0) | 0(0.0) |  |
| **The most impressive touches of death, n (%)** |  |  |  | 0.258^a^ |
| Parents | 29(72.5) | 14(70.0) | 15(75.0) |  |
| Spouse | 4(10.0) | 1(5.0) | 3(15.0) |  |
| Brothers and sisters | 4(10.0) | 2(10.0) | 2(10.0) |  |
| Other | 3(7.5) | 3(15.0) | 0(0.0) |  |

*Note.* a, χ2 test; b, Fisher's exact test.

Additional file 6 Participant Characteristics and Group Comparisons (Continued).

| Characteristics | Total  (N=40) | Intervention group(N=20) | Control group  (N=20) | *t*/*χ*^2^ (*P*) |
| --- | --- | --- | --- | --- |
| **Ever talk about death, n (%)** | | | | 0.542^a^ |
| No | 25(62.5) | 14(70.0) | 11(55.0) |  |
| Open and natural | 10(25.0) | 4(20.0) | 6(30.0) |  |
| Relaxed, but ended quickly | 1(2.5) | 1(5.0) | 0(0.0) |  |
| Only when necessary, and avoid someone | 3(7.5) | 1(5.0) | 2(10.0) |  |
| Try to avoid | 1(2.5) | 0(0.0) | 1(5.0) |  |

*Note.* a, *χ*^2^ test.

Additional file 6 Participant Characteristics and Group Comparisons (Continued).

| Variables | Intervention group(N=20) | Control group(N=20) | *t*/*χ*^2^ (*P*) |
| --- | --- | --- | --- |
| **DAP-R** |  |  |  |
| Fear of death | 14.30±3.51 | 14.65±4.61 | 0.789^a^ |
| Death avoidance | 15.65±4.30 | 12.85±3.71 | 0.034^a^ |
| Neutral acceptance | 22.00（20.00,25.00） | 20.00（20.00,23.75） | 0.186^b^ |
| Approach acceptance | 21.00（20.00,27.25） | 22.00（20.00,29.75） | 0.723^b^ |
| Escape acceptance | 15.50（12.00,19.00） | 12.50（11.25,16.00） | 0.127^b^ |
| Total score | 90.50±11.06 | 86.55±12.50 | 0.297^a^ |
| **PIL** |  |  |  |
| Quality of life | 21.00（17.50,23.00） | 20.00（16.25,21.00） | 0.549^b^ |
| Value of life | 25.00（23.50,27.00） | 24.00（20.00,26.00） | 0.151^b^ |
| Goal of life | 27.35±4.12 | 26.40±4.19 | 0.475^a^ |
| Freedom of life | 8.00（6.00,9.00） | 6.00（5.00,7.00） | 0.017^b^ |
| Total score | 79.15±12.28 | 75.10±10.18 | 0.263^a^ |

*Note.* a, t-test; b, Wilcoxon rank sum test; DAP-R, Death Attitude Profile Revised; PIL, Purpose in Life Test.

Additional file 7 Death Attitude and Meaning of Life of Two Groups

After the Intervention.

| Variables | Intervention group | Control group | *t*/*z* | *P*^a^ | *P*^b^ |
| --- | --- | --- | --- | --- | --- |
| **DAP-R** |  |  |  |  |  |
| Fear of death | 12.55±4.32 | 15.15±2.20 | -2.39 | 0.002 | 0.028 |
| Death avoidance | 15.65±4.29 | 16.10±3.56 | -0.36 | 0.237 | 0.432 |
| Neutral acceptance | 23.50（20.00,25.00） | 20.50（20.00,23.75） | -1.26 | 0.207 | 0.106 |
| Approach acceptance | 24.50（20.00,31.00） | 22.00（20.00,30.00） | -0.13 | 0.891 | 0.726 |
| Escape acceptance | 16.50（14.00,20.00） | 18.00（12.00,18.00） | -0.81 | 0.413 | 0.762 |
| Total score | 91.55±11.52 | 92.70±10.19 | -0.33 | 0.528 | 0.455 |
| **PIL** |  |  |  |  |  |
| Quality of life | 22.00（19.25,23.00） | 19.00（18.00,20.00） | -2.51 | 0.012 | 0.141 |
| Value of life | 28.00（26.00,29.75） | 23.50（22.00,25.75） | -3.34 | 0.001 | 0.031 |
| Goal of life | 29.85±3.87 | 27.30±3.62 | 2.15 | 0.038 | 0.035 |
| Freedom of life | 8.00（6.25,9.00） | 6.00（5.00,7.00） | -3.38 | 0.001 | 0.003 |
| Total score | 85.50±12.21 | 76.15±8.54 | 2.80 | 0.008 | 0.017 |

*Note.* a, t test or Wilcoxon rank sum test; b, Multiple regression; DAP-R, Death Attitude Profile Revised; PIL, Purpose in Life Test.


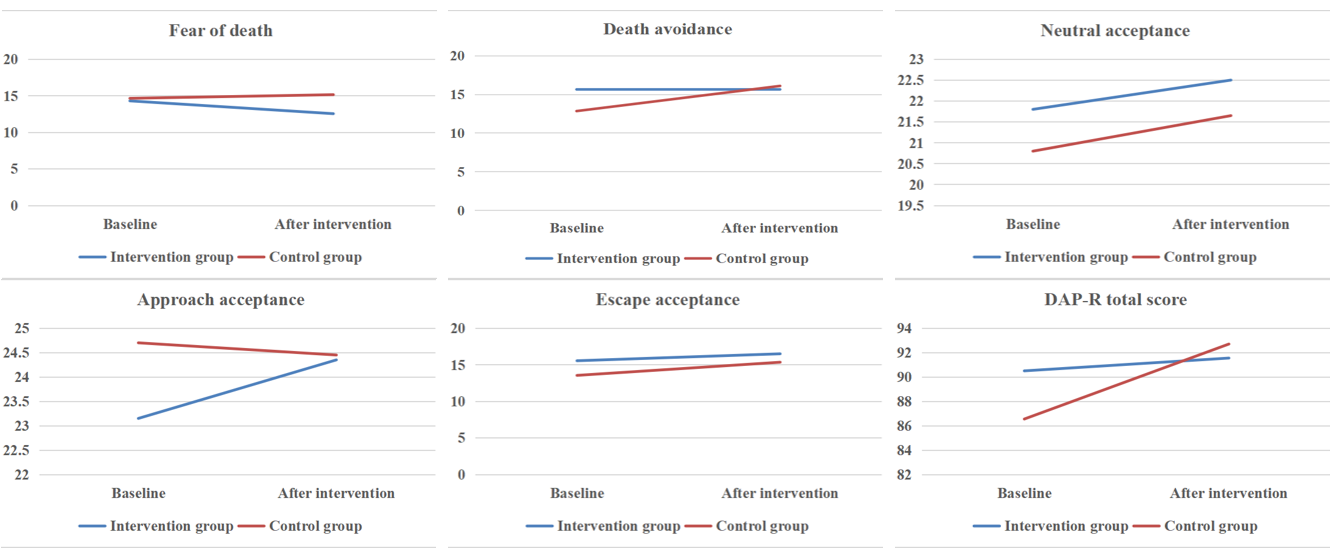


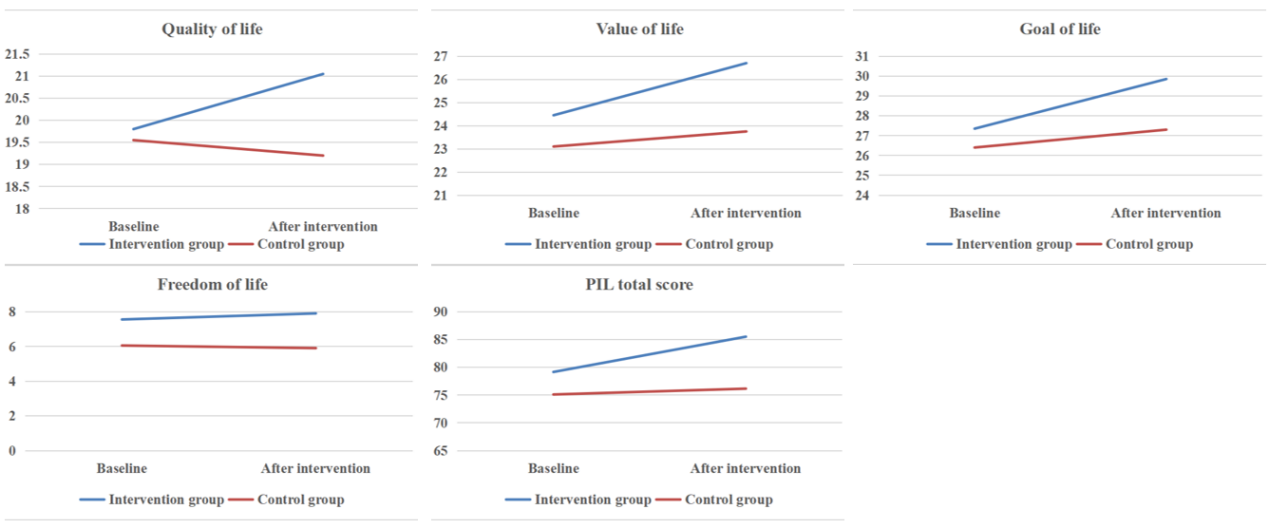


Additional file 8 The Trend of Death Attitudes and Meaning of Life
